# The Prevalence of Urinary Tract Infections and Antibiotic Prescription Treatments Across Three Countries: A Retrospective Study Using an Electronic Medical Record

**DOI:** 10.7759/cureus.46466

**Published:** 2023-10-04

**Authors:** Rachael Lee, Nathan P Hass, Allie Kollitz, Meghan Wilson

**Affiliations:** 1 Medicine, Edward Via College of Osteopathic Medicine, Blacksburg, USA; 2 Biological Sciences, Virginia Polytechnic Institute and State University, Blacksburg, USA; 3 Biochemistry, Virginia Polytechnic Institute and State University, Blacksburg, USA; 4 Biology, Edward Via College of Osteopathic Medicine, Blacksburg, USA

**Keywords:** antibiotics, honduras, el salvador, dominican republic, emr, utis, credo

## Abstract

Introduction

The Clinical Rotation Evaluation and Documentation Organizer (CREDO) is an electronic medical record (EMR) system created by the Edward Via College of Osteopathic Medicine (VCOM). International healthcare providers who partner with VCOM can gain access to CREDO and input their patient data. The objective of this study was to determine the frequency of urinary tract infection (UTI) diagnoses and prescription use over a one-year period in three Latin American countries.

Methods

Researchers analyzed the frequency of UTI diagnosis with corresponding prescription recommendations over a 12-month period in three Latin American countries (i.e., Dominican Republic, El Salvador, and Honduras) that utilize the CREDO system. For each month between May 2021 and May 2022, the total number of UTI diagnosis codes and prescription codes were summed, graphed, and analyzed.

Results

In El Salvador, there were 142 UTIs and 126 corresponding prescriptions written for UTIs reported from May 2021 to May 2022 but diagnoses were not consistent each month. Ciprofloxacin was prescribed most frequently at a rate of 43.7% in El Salvador. In Honduras, there were 68 UTIs and 68 corresponding prescriptions written for the UTIs reported from May 2021 until May 2022 with Ciprofloxacin being prescribed most frequently at a rate of 39.7%. Again, diagnosis frequency was not consistent each month. In the Dominican Republic, there were 42 UTIs and 14 corresponding prescriptions written for those UTIs reported, however, data only reflected two months' worth of UTI diagnoses from May 2021 to May 2022. Fosfomycin was prescribed most frequently at a rate of 61.5%.

Conclusion: The findings above suggest that there are inconsistent UTI reports throughout the year with a varied use of antibiotics prescriptions for UTIs. The discovered discrepancies in disease reporting, or lack thereof of reporting, and prescription recommendation suggest inconsistent reporting in CREDO. In the future, focused education or revision on CREDO reporting and uniform coding practices could be implemented to reduce these inconsistencies.

## Introduction

Urinary tract infections (UTIs) are bacterial infections affecting the urinary tract. The bacteria enters the urethra and travels up the urinary tract, oftentimes causing an infection in the bladder, known as an ascending UTI. If the infection continues up the ureter, it can reach the kidney and cause a more serious infection [1]. When damage is done to the kidney via infection, many health complications can develop due to renal scarring. These include hypertension, IgA nephropathy, renal vasculitis, lupus nephropathy, and renal failure [2-3]. These complications can cause long-term damage, thus affecting the overall health of the individual and possibly even causing death if left untreated. UTIs are often a result of poor hygiene around and on the genital area. This may be due to but not limited to unclean undergarments, washing or bathing conditions, and unsafe sexual practices.

UTIs should be treated with antibiotics to control the spread to other systems of the body, creating more serious infections. Antibiotics including nitrofurantoin monohydrate/macrocrystals, trimethoprim-sulfamethoxazole (TMP-SMX), fosfomycin, and pivmecillinam are commonly used as pharmaceutical therapies for UTIs [4]. However, with the rise of antibiotic-resistant strains, other therapies are in development for UTIs. Some of these new developing therapies include vaccines against virulence factors, mannosides, and pilicides [5].

An electronic medical record (EMR) is an online database used by providers to efficiently and safely track conditions, diagnoses, and treatments of individuals. EMRs can also provide quantitative information which can be used by professionals to track epidemics, antibiotic stewardship, and the prevalence of antibiotic-resistant strains. The Clinical Rotation Evaluation and Documentation Organizer (CREDO) is an EMR system created by the Edward Via College of Osteopathic Medicine (VCOM). This system was developed to track patient data in a similar system that students use outside of their clinical rotations. It uses the International Classification of Diseases (ICD) to code for items as designated by the World Health Organization. CREDO allows space for doctors to insert free-text notes on patients while collecting a minimal amount of personally identifying data. No data is collected that could be identifying health information or that could breach the Health Insurance Portability and Accountability Act [6]. VCOM has partnerships with local healthcare providers, as well as providers worldwide, who have access to and utilize this EMR system. This is especially useful in areas where medical records are still completed by hand or in areas that do not have a centralized medical record system. Data reported within CREDO can then be viewed by researchers to look for general trends and patterns of disease diagnosis, diagnosable conditions, prescription use, etc., within and across clinics and countries.

Honduras, El Salvador, and the Dominican Republic are three Latin American countries with access to medical reporting in the CREDO database through established partnerships with VCOM. This study used data within the CREDO database to provide a brief overview of UTI frequency and prescription use rates for UTIs occurring at CREDO-utilizing clinics in these countries. Documenting the rate at which and type of antibiotics that are prescribed for UTIs gives healthcare providers a better understanding of the consistencies and discrepancies between UTI diagnosis and prescription use in different countries. Ideally, there should be a consistent diagnosis of UTIs each month and an equivalent number of prescriptions to treat the diagnosed UTIs as these infections are not self-regulating. The objective of this study was to determine the frequency of UTI diagnoses, over a one-year period, with prescription usage in each of the three countries.

## Materials and methods

This study and the methods involved were reviewed and approved by the VCOM Institutional Review Board (RECORD #: 2022-038, effective date: June 16, 2022). To begin the retrospective data analysis, the entire dataset was first downloaded from the CREDO database using the “Export to Microsoft Excel” function. The dataset included every single entry recorded in each clinic for the dates between May 2021 to May 2022 in El Salvador, Honduras, and the Dominican Republic. The Excel document contained all of the data for each patient seen from every clinic within a selected country during this time frame (e.g., patient identification, diagnosis, prescription recommendation, etc.). The filter function was then utilized within Excel to extract datasets for clinics located in the Dominican Republic, Honduras, and El Salvador, respectively, during the May 2021 to May 2022 timeline, leaving three individual datasets to analyze.

Data extraction continued within each country's dataset, individually. Inclusion criteria for patient data had to contain the keyword “Urinary” in the “Description” section of the entry. From those included entries, all the prescriptions given and other diagnoses for the patient on that day were also retained. 

The International Classification of Diseases, Tenth Version (ICD-10) code for the disease or ailment must have read as N39.0 (“Urinary tract infection, site not specified”), N39 (“Other disorders of urinary system”), or O86.20 (“Urinary tract infection following delivery, unspecified”). The ICD-10 is the classification system used by CREDO to give an identifying code to each disease or diagnosis [7]. This allowed for streamlined reporting of ailments and a systematic way to identify the disease each patient had or was diagnosed with [7] during the 12-month period. If the patient did not code for at least one of these three, the data were excluded. Once patients with one of the specified ICD10 codes were identified, the prescriptions given to that patient were then analyzed. The prescription codes that were isolated are found in Table 1. 

**Table 1:** The prescription codes isolated during data collection and the medications that they code for.

Code	Generic Name	Brand Name
A01AB17	Metronidazole	Mnz
A01AB18	Clotrimazole	Mycelex
A02BD10	Lansoprazole, amoxicillin, and levofloxacin	Prevacid
B01AC06	Acetylsalicylic acid	Aspirin
C10AB08	Ciprofibrate	Modalim
D06BX01	Metronidazole	Mnz
G01AA10	Clindamycin	Cleocin
G01AF01	Metronidazole	Flagyl
G04BX06	Phenazopyridine	Azo
J01CA04	Amoxicillin	Moxatag
J01CR02	Amoxicillin and enzyme inhibitor	Augmentin
J01DB01	Cefalexin	Keflex
J01DD04	Ceftriaxone	Rocephin
J01DD08	Cefixime	Suprax
J01EA01	Trimethoprim	Proloprim
J01EC01	Sulfamethoxazole	Smz
J01EE01	Sulfamethoxazole and trimethoprim	Bactrim
J01EE04	Sulfamoxole and trimethoprim	
J01FA09	Clarithromycin	Biaxin
J01FA10	Azithromycin	Zithromax
J01GB03	Gentamicin	Gentak
J01GB06	Amikacin	Amikin
J01MA02	Ciprofloxacin	Cetraxal
J01MA12	Levofloxacin	Levaquin
J01XE01	Nitrofurantoin	Macrobid
J01XX01	Fosfomycin	Monurol
P01AC01	Diloxanide	—
P02CA03	Albendazole	Albenza
S01AE05	Levofloxacin	Levaquin

## Results

El Salvador

**Figure 1:**
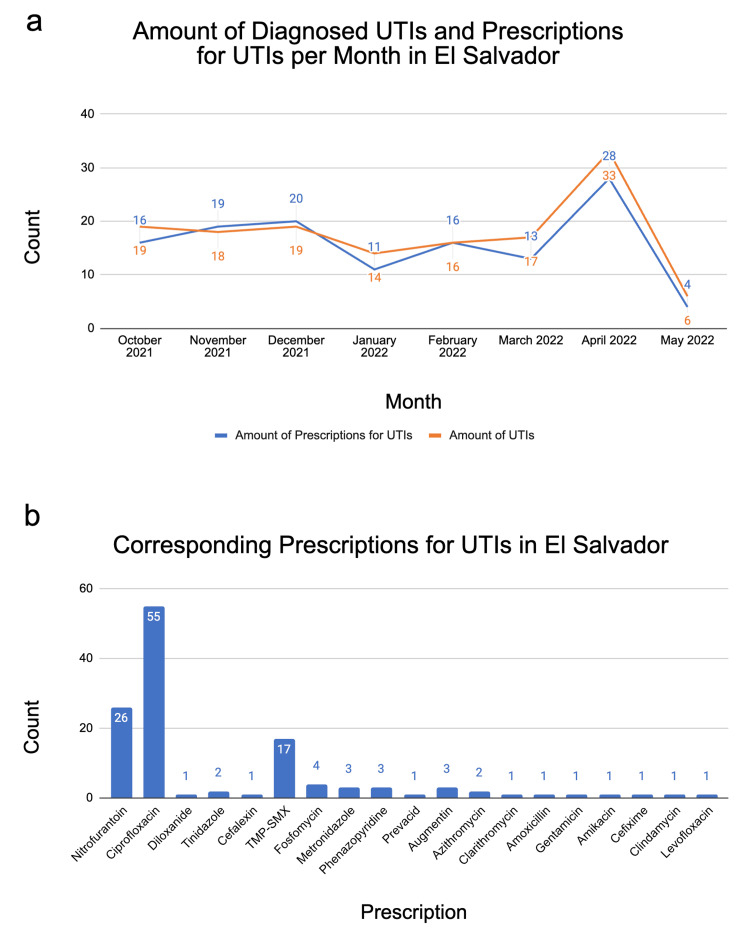
Data collected for El Salvador. (a) The line graph depicts the diagnosed UTIs (orange line) per month, overlaid with the number of prescriptions given (blue line) to treat UTIs. The data in the graph are shown over a period of October 2021 to May 2022. From May 2021 to September 2021, there were no documented UTI diagnoses or prescriptions for UTIs given, so those months were omitted from the graph. (b) The bar graph shows the different medications prescribed for UTIs and the frequency at which they were prescribed over the same period of October 2021 to May 2022.

In El Salvador, 142 UTIs and 126 prescriptions for UTIs were reported from May 2021 to May 2022 (Figure 1a). This represents data from one CREDO-using clinic in El Salvador. Diagnosis reports of UTIs were inconsistent over the 12-month period, with five months void of any reported UTI diagnoses or prescription use (i.e., May, June, July, August, and September of 2021). Of the months with reported UTIs, the lowest-reporting month was May 2022 (n=6) in comparison to the highest-reporting month, April 2022 (n=33). In the eight months shown, prescription frequency did not match UTI diagnosis frequency (Figure 1a). Ciprofloxacin was prescribed the most frequently, accounting for 43.65% of all reported UTI prescriptions for El Salvador from May 2021 to May 2022 (Figure 1b).

Honduras

**Figure 2:**
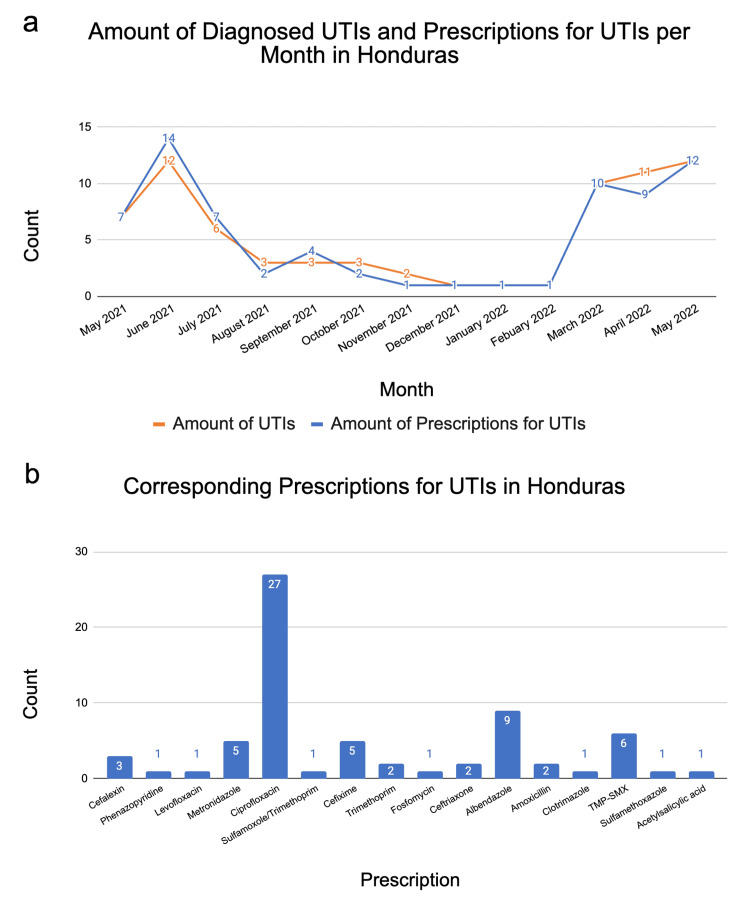
Data collected for Honduras. (a) The line graph depicts the diagnosed UTIs (orange line) per month, overlaid with the number of prescriptions given (blue line) to treat UTIs. The data in the graph are shown over a period from May 2021 to May 2022. From December 2021 to February 2022, there were no documented UTI diagnoses or prescriptions for UTIs given. (b) The bar graph shows the different medications prescribed for UTIs and the frequency at which they were prescribed over the same period of May 2021 to May 2022.

In Honduras, there were 68 reported UTIs and 68 prescriptions for UTIs from May 2021 to May 2022; however, the prescription use frequency did not correspond to the UTI reports in each month (Figure 2a). This represents data from one CREDO-using clinic in Honduras. There were three months void of UTI reports (i.e., December 2021, January 2022, and February 2022). Of the months with reported UTIs, the lowest-reporting month was November 2021 (n=2) in comparison to the highest-reporting month of June 2021 (n=12) (Figure 2a). A total of 17 different medications were prescribed for UTIs. Ciprofloxacin was prescribed the most frequently, accounting for 39.7% of all reported UTI prescriptions for Honduras from May 2021 to May 2022 (Figure 2b).

Dominican Republic

**Figure 3:**
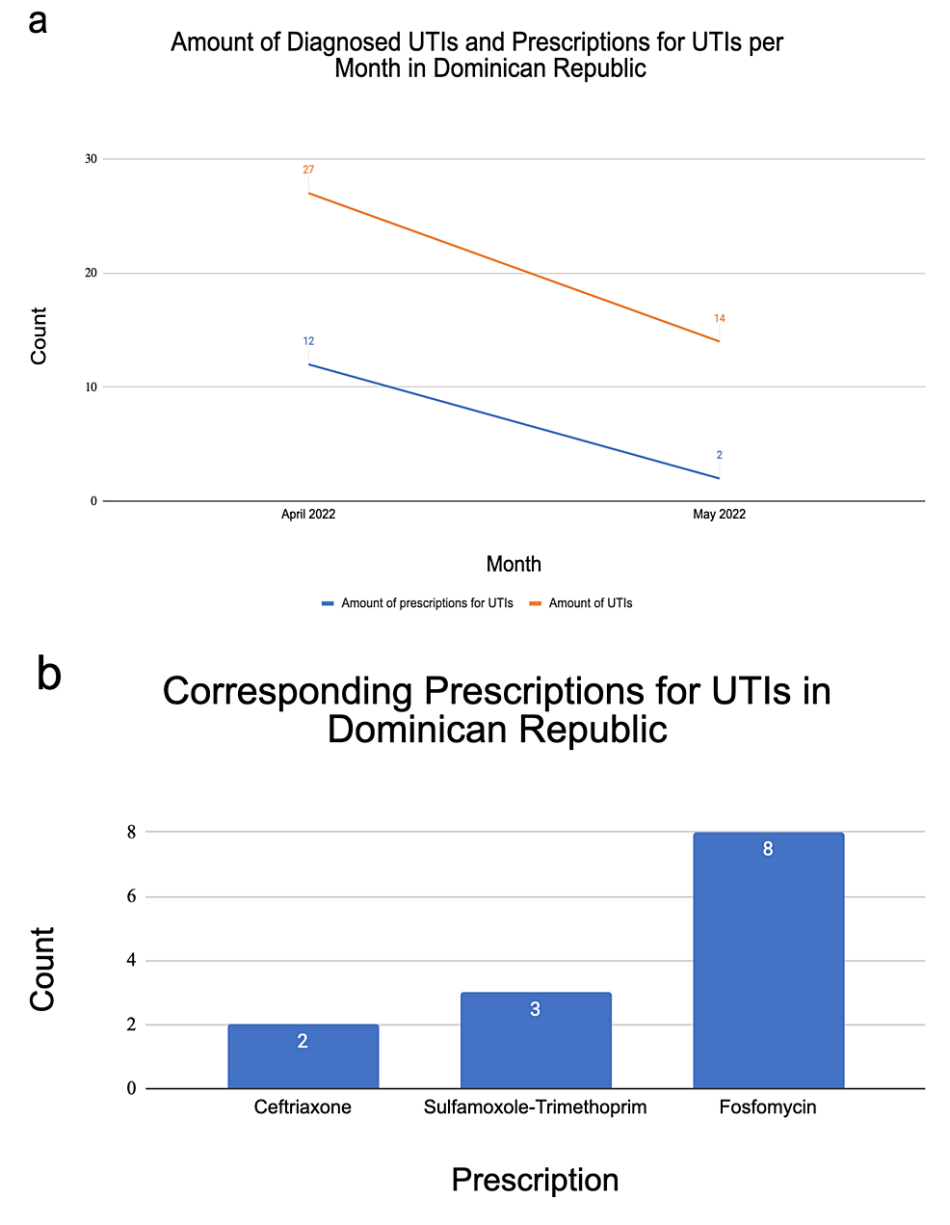
Data collected for the Dominican Republic. (a) The line graph depicts the diagnosed UTIs (orange line) per month, overlaid with the number of prescriptions given (blue line) to treat UTIs. The data in the graph are shown over a period from April 2022 to May 2022. From May 2021 to March 2022, there were no documented UTI diagnoses or prescriptions for UTIs given, so those months were omitted from the graph. (b) The bar graph shows the different medications prescribed for UTIs and the frequency at which they were prescribed over the same period of April 2021 to May 2022.

In the Dominican Republic, there were 41 reported UTIs and 14 prescriptions used for UTIs reported from May 2021 to May 2022 (Figure 3 a) from three total CREDO-using clinics in the Dominican Republic. The months of April 2022 and May 2022 were the only months that codes for a UTI were documented in the CREDO database. All other months during this timeframe had no UTI codes reported or documented. Three different medications were prescribed for UTIs (Figure 3 b). Fosfomycin was prescribed the most frequently, accounting for 61.5% of all reported UTI prescriptions for the Dominican Republic from May 2021 to May 2022 (Figure 3 b).

## Discussion

In CREDO, UTIs were documented infections in El Salvador, the Dominican Republic, and Honduras. The prescribing practices for each country involved a variety of antibiotics. The objective of this study was to determine the frequency of urinary tract infection diagnoses and prescriptions used over a one-year period in each of the three countries.

It is important to note that there were discrepancies in the data for each country. In all three countries, the rate at which prescriptions were used to treat UTIs did not equal the number of reported UTIs in each month (Figures 1a, 2a, 3a). There were several months in Honduras and El Salvador with zero reports of UTIs, but the use of prescriptions for UTIs were reported (e.g., January 2022 in Honduras, Figure 2a). In other cases, UTIs were reported at a higher frequency than prescriptions were reported. For example, in April 2022 in El Salvador, there were 33 reported UTIs and only 28 reported prescriptions, Figure 1a. Additionally, in El Salvador, there was an unexplainable increase in the number of diagnosed UTIs and prescriptions for UTIs per month in April 2022. Another finding was that there were no reported UTI or prescription entries from December 2021 to February 2022 for CREDO-reporting clinics in Honduras. The most noticeable discrepancy in the frequency of UTI reports was found within the Dominican Republic. Over the course of a 12-month period, there were only reports of UTI incidents during the months of April 2022 and May 2022 (Figure 3a). These discrepancies could indicate a variety of scenarios: 1. the same UTI is being treated with multiple prescriptions, 2. the UTIs are left untreated, or 3. there are errors in the coding practices for UTIs or prescription usage within CREDO. 

The reasons for the discrepancies listed above are not currently known. According to a study conducted at local health stations in six towns in Bonao County, Dominican Republic, there are many barriers to successfully implementing EMR use in developing countries [8]. Some of these barriers included a lack of EMR infrastructure and little technical expertise among healthcare providers. Implementing specific interventions tailored to physician’s needs was directly correlated with improved EMR use [8]. This could be a solution for improving reporting practice consistency among CREDO-reporting clinics in El Salvador, Honduras, and the Dominican Republic. Another study utilizing HospiScope data from nine Latin American nations found that EMR success depends on not only human factors but also regulatory, legal, financial, technological, and organizational factors [9]. More specifically, it is important to consider a country’s core cultural values when discussing barriers to EMR adoption and implementation [9]. The discovered discrepancies in UTI reporting and coding in CREDO, particularly in the Dominican Republic, imply there is a lack of consistent UTI reporting and the need for education or revision of these practices. These data justify the need for further investigation to determine the exact reporting discrepancies within countries. Additionally, these gaps in CREDO data show a need to investigate the true source of the missing data to accurately fix and improve this situation.

When addressing the seasonality of UTIs, there are data that support hot weather as a risk factor for UTIs [10]. Our data was not consistent with this data, as the hotter months did not report the highest recorded numbers of UTIs. For example, in Honduras, fewer UTIs were reported in July and August than in typically cooler months such as March, April, and May. The explicit reasoning for this is not known and is worth investigating further in the future. 

When considering treatment for acute simple UTIs, first-line empiric therapy includes nitrofurantoin monohydrate/macrocrystals, trimethoprim-sulfamethoxazole (TMP-SMX), fosfomycin, and, if available, pivmecillinam [4]. There are pros and cons to the utilization of each medication, so the proper medication should be chosen based on individual health circumstances. If beta-lactams cannot be used, fluoroquinolones such as ciprofloxacin or levofloxacin may be used alternatively [4]. In the Dominican Republic, fosfomycin was the most commonly prescribed antibiotic for UTIs. This was consistent with the previously mentioned first-line empiric therapies used for acute simple UTIs. However, in El Salvador and Honduras, ciprofloxacin was the most commonly prescribed medication for UTIs. This suggests that in El Salvador and Honduras, fluoroquinolones were considered first-line empiric therapy. According to a study done on antibiotic sensitivity in UTIs across Central America, fosfomycin was found to be the most effective out of 47 antibiotics used [11]. The same study concluded that TMP-SMX had the highest percentage of resistance out of 47 antibiotics used [11]. The aforementioned results aligned with the researchers’ findings that TMP-SMX was not the antibiotic of choice for UTIs in El Salvador, Honduras, or the Dominican Republic and that fosfomycin was an antibiotic of choice in the Dominican Republic.

Overall, El Salvador, Honduras, and the Dominican Republic each favored the use of different medications to treat UTIs. This preference for fluoroquinolone use in El Salvador and Honduras could be useful when analyzing antibiotic stewardship to effectively treat infections and combat antibiotic resistance in these countries [12]. This study provided more insight into antibiotic prescribing practices throughout various clinics in El Salvador, Honduras, and the Dominican Republic. Additionally, it was important to note the potential variation in antimicrobial resistance, as it corresponds to the influenza season. One study done on 257 U.S. healthcare institutions found that the influenza season was correlated to an increase in antibiotic-resistant gram-negative pathogens [13]. Although this study did not include Latin American countries, it provided applicable awareness of the need to be cautious of increased antimicrobial resistance following the influenza season. More research on antibiotic prescribing practices may provide a better understanding of antibiotic stewardship practices in these developing countries and ways to improve them.

There were a few limitations to the study that were worth noting. First, when deciding which diagnostic codes to include in the study, it was determined that only listings that specifically stated that a urinary tract infection was being diagnosed would be included in the study. However, this excluded diagnostic codes containing “Urinalysis,” which indicated that testing had been done on the urine. In many cases, reports of urinalysis were indicated, and prescriptions were suggested, but there was no confirmation of UTI diagnosis. Data from those circumstances were not included in our frequency report. Another limitation was that historically, the medications found in Table 1 have been used to treat urinary tract infections, so the researchers inferred that these prescriptions were given to these patients for that reason [14]. Many of the patients were linked to multiple diagnosable codes and multiple corresponding medications. However, there was not a direct linkage between which medication was prescribed for which diagnosis. This can be seen as a limitation as it was difficult to confirm that the patient was receiving the particular prescription medication for the diagnosis of a UTI or for another comorbid diagnosis.

## Conclusions

These data, within each country, suggested that there were inconsistent UTI reports throughout the year, and it is unclear if all UTI reports are linked with antibiotic treatment. The variability in the number of UTI reports and prescription frequency was seen in each country. There was, however, consistency in the type of antibiotics prescribed for reported UTIs in El Salvador and Honduras but not in the Dominican Republic. The discovered discrepancies in disease reporting and prescription recommendation suggest inconsistent reporting in CREDO. It is evident that there is inconsistent reporting in CREDO, but it is unclear why these inconsistencies exist (e.g., system infrastructure, cultural differences, disparities, access to diagnostic tools and medications, etc.). Further research is needed to understand why there are discrepancies in disease reporting and prescription use. A greater understanding of those factors can lead to the development of strategies that may improve healthcare systems in underserved areas.
